# Risk Factors Associated with Hemoparasites in Dual-Purpose Cattle of Colombia

**DOI:** 10.3390/pathogens14010062

**Published:** 2025-01-12

**Authors:** César A. Murcia-Mono, Sergio Falla-Tapias, Andrés F. Morales Cabrera, Laura C. Navia Álvarez, Leidy Rivera-Sánchez, Yolanda Gómez Vargas, William O. Burgos-Paz

**Affiliations:** 1 Semillero de investigación CIETVET, Facultad de Medicina Veterinaria y Ciencias Afines, Programa de Medicina Veterinaria y Zootecnia, Corporación Universitaria del Huila CORHUILA, Neiva 410010, Colombia; cesar.murciam@corhuila.edu.co (C.A.M.-M.); sergio.falla@corhuila.edu.co (S.F.-T.); afmorales-2020b@corhuila.edu.co (A.F.M.C.); lab.sanitario@corhuila.edu.co (L.C.N.Á.); 2 Departamento de Biología de la Reproducción, Universidad Autónoma Metropolitana, Iztapalapa 09040, Ciudad de México, Mexico; ydiel_@hotmail.com; 3 Centro de Investigación Tibaitatá, Corporación Colombiana de Investigación Agropecuaria AGROSAVIA, Mosquera 250047, Colombia; ygomez@agrosavia.co; 4 Centro de Investigación Turipaná, Corporación Colombiana de Investigación Agropecuaria AGROSAVIA, km 13 vía Montería, Cereté 230550, Colombia

**Keywords:** vector-borne diseases, hemoparasites, prevalence, risk factors, herd management

## Abstract

Hemoparasitic diseases represent a significant problem with a considerable impact on tropical and subtropical areas of the world. These conditions cause economic losses associated with multi-organic failure and even the death of animals. In these areas, the hemoparasites are transmitted in an enzootic cycle when infectious cattle, such as persistently infected animals, including cows, contribute to the success of transmission. However, the factors associated with transmission have always been considered environmental issues, disregarding herd management and practices. In this sense, we conducted a cross-sectional study sampling 360 female cattle older than one year to identify infectious cattle using the PCR technique. We employed a dichotomic questionnaire for association analyses in 150 herds of the southern Andean region of Colombia. Overall prevalence with infectious cattle was 52.5% for *Babesia* spp., *Anaplasma* spp., and *Trypanosoma* spp., and the significant risk factors (*p* < 0.05) included geographic area, animal weight, purchase of cattle for fattening, disinfection of clothing after contact with neighboring animals, self-medication, separation of animals in pens, supply of mineralized salt, presence of livestock from other owners on the farm, prevention of joint trauma, documented milking routine, and sending blood samples for analysis. These practices permitted the maintenance of persistently infected animals and their movement to shed the agents to other animals in the presence of vectors. This suggests the importance of implementing comprehensive control and training measures to reduce the infectious cattle and, therefore, the profitability of dual-purpose livestock farms in the Andean region of southwestern Colombia.

## 1. Introduction

In tropical and subtropical areas, vector-borne pathogens cause significant economic losses due to hemoparasitism in cattle. Ixodid ticks and biting flies, which show similar distribution patterns across many regions worldwide, enhance the transmission of primary hemoparasitic agents, such as *Babesia*, *Anaplasma*, and *Trypanosoma* [1]. The spread of these pathogens is influenced by various epidemiological factors related to production systems and herd management practices, leading to reduced productivity, increased veterinary losses, and economic impacts [2]. Persistently infected cattle play a crucial role in sustaining the transmission cycle, with their presence associated with husbandry practices and environmental changes [3]. In South American countries, including Colombia, stable to unstable enzootic conditions with differing prevalence rates can predispose herds to ongoing transmission cycles or outbreaks when infected cattle are present [4].

Diseases of national interest, according to Resolution 003714 of the Instituto Colombiano Agropecuario ICA, include babesiosis, anaplasmosis, and trypanosomiasis due to their significant impact on livestock health and productivity [5]. These diseases primarily cause anemia, along with weight loss, increasing mortality, and making individuals more susceptible to other diseases [6]. Economic losses due to deaths from these diseases were estimated at approximately USD 108,000, broken down as follows: anaplasmosis, USD 46,057; babesiosis, USD 24,721; trypanosomosis, USD 34,881, and cases with more than one hemoparasite, USD 2370 [7].

In mountainous regions, herds are exposed to significant variations in rainfall and climate, including differences in temperature and humidity based on altitude. Extensive and semi-extensive livestock management practices, coupled with limited strategic interventions and best practices under changing climatic conditions, promote the proliferation of hematophagous arthropods and the persistence of infected cattle [8]. Herds in regions experiencing co-infestation by ectoparasites exhibit diverse epidemiological scenarios involving pathogens such as *Babesia bovis*, *Babesia bigemina*, *Anaplasma marginale*, *Trypanosoma vivax*, and *Trypanosoma evansi* as single or mixed infections [5,9,10]. Traditionally, these pathogens’ epidemiological dynamics were studied in limited areas, often overlooking their impact across broader geographic regions.

Therefore, the aim of this study was to determine the prevalence of these diseases in dual-purpose cattle in Colombia, providing insights into the various management practices employed across 150 herds comprising 360 dual-purpose female cattle in the southwestern Andean region, which are critical for mitigating economic losses and ad-dressing potential epidemiological challenges associated with hemoparasites in these operations. Additionally, the study aims to identify and evaluate specific risk factors contributing to the occurrence of hemotropic infections, thereby supporting the protection of regional interests within the *One Health* framework [11,12].

## 2. Materials and Methods

### 2.1. Study Area

The study was conducted in 24 municipalities within the Huila department, located in the southwestern Andean region of Colombia. The study was distinguished by the thermal floor diversity, encompassing climates ranging from warm to cold-humid [13]. The region exhibits a bimodal rainfall pattern, with two distinct wet seasons occurring from March to May and October to December. The annual precipitation ranges between 1500 and 2000 mm. The region experiences two distinct dry seasons, one from January to February and the other from July to August [14]. The number of days with precipitation varies between 100 and 150 in the Magdalena Valley, although less than 100 can be recorded in parts of the municipalities of Aipe and Villavieja. In the foothills, rainy days increase slightly, reaching 200 or more in isolated locations south of the department. These areas experience high humidity, with average temperatures ranging from 26 to 28 degrees Celsius [13].

### 2.2. Study Population

To identify risk or protection factors against diseases, initially, information on 230 variables about animal management and epidemiological practices was collected on each herd, addressing the following categories: good livestock practices (GLP), animal health, feeding, reproduction, facilities, veterinary medication, personnel, clinical history, sanitation, transportation, traceability, biosecurity, and socioeconomic factors. The sample size was calculated, including the official records of cow vaccination programs, to be 2618 female cattle from 224 farms with an expected prevalence of 50% for hemoparasites as identified by Ríos-Tobón et al. (2014) in similar cattle management [15]. A confidence interval of 95% and a design effect of 5% was used. The data were processed using Epi Info™ v 7.2.0.1 software (CDC, 2016), which estimated a sample size of 385 animals. However, the final sample size was 360 cows from 150 farms, sampled between May and June 2023 to account for potential field losses. During the sampling period, the animals were exposed to weather conditions typical of the final phase of the first humid period and the transition from the dry to the rainy season. These conditions were characterized by high levels of water evaporation, which created suitable conditions for ixodid tick development and less favorable conditions for biting flies that develop in the rainy season.

### 2.3. Processing Samples and DNA Extraction

Given the vector-borne transmission and the presence of infectious cattle, blood was collected from the tail vein in EDTA tubes and stored at 4 °C. For DNA extraction, a DNA2000 kit (Corpogen, Colombia, ref: BM-001) was used. 250 μL of blood was transferred to a 1.5 mL microcentrifuge tube, followed by 25 μL of Proteinase K. Then, 250 μL of BLU buffer was added and vortexed until the solution was homogenized. The solution was then incubated at 55 °C for 15 min. Then, 250 μL of ethanol (96–100%) was added and mixed vigorously. The minispin column was then placed in a collection tube, and the mixture was transferred by pipetting. It was centrifuged at 8000 rpm for 1 min, and the contents of the collection tube were discarded. The minispin column was then placed in a new collection tube, and 500 μL of WB1 buffer was added. The sample was centrifuged at 8000 rpm for 1 min, and the contents of the collection tube were discarded. The minispin column was then placed in a new collection tube, and 500 μL of WB2 buffer was added. The two previous procedures were then repeated, but this time, 800 μL of WB2 buffer was added, centrifuged at 8000 rpm for 1 min, and the contents of the collection tube discarded. The minispin column was then centrifuged at maximum speed for 3 min to dry. The minispin column was then placed in a new 1.5 μL labeled microcentrifuge tube, and 100–200 μL of EB buffer was added. The tube was sealed and incubated for 2 min at room temperature. Finally, it was centrifuged at maximum speed for 1 min to elute the genomic DNA, which was purified and stored at 2–8 °C for a few days.

### 2.4. PCR Technique

DNA samples were processed by endpoint polymerase chain reaction (PCR) using Taq DNA Polymerase Master Mix 2X (Invitrogen, ThermoFisher, Waltham, MA, USA, including the primer sequences in Table 1. The PCR reaction volume was 12.5 µL using Taq red mix bioline 6.25 µL, 0.5 µL each of forward and reverse primers, and 2 µL DNA (50 ng/µL). For the negative control, water was added instead of DNA. Positive and negative controls were included in each analysis. PCR was performed on a Veritu-96 Well Thermal Cycler (ThermoFisher, Waltham, MA, USA), following an initial denaturation at 95 °C for 1 min, then 35 cycles of denaturation (95 °C for 15 s), annealing (58 °C for 15 s), and extension (72 °C for 10 s). There was one final extension cycle at 72 °C for 1 min and cooling at 4 °C. A 143 pb, 100 pb, and 298 pb for *Anaplasma* spp., *Babesia* spp., *and Trypanosoma* spp. products were observed by electrophoresis on 1.5% agarose gel with a cyber green stain.

### 2.5. Data Analysis

The prevalence was calculated using a 95% confidence interval (CI) with data entry. Data from an epidemiological questionnaire with dichotomous responses were used for association analysis, using Pearson’s correlation to analyze the relationships between the different pathogens. A descriptive study of risk factors was then performed. Binary logistic regression models were then used, where the dependent variable was the result of the PCR tests (0: negative, 1: positive for some hemoparasites), and the risk factors were defined as independent variables. For each factor, odds ratios (ORs) and 95% confidence intervals were analyzed to determine the influence of the predictive variables on the positive or negative test result (*p*-value < 0.05). To visually identify the relationships between the factors and the animal management activities carried out in the herds, risk factors with a significant effect on the results of the presence or absence of the disease were analyzed using Euclidean distance and hierarchical clustering. Descriptive analyses, logistic regression models, and risk factor analyses were performed using the R statistical program 4.3.3 (R Core Team, 2024).

## 3. Results

### 3.1. Prevalence

Of the analyzed samples, 52.5% (189/360) were positive for hemoparasites. Among these, the most prevalent pathogen was *Trypanosoma* spp., followed by *Babesia* spp. and, finally, *Anaplasma* spp. Conversely, co-infections exhibited a prevalence that varied slightly compared to individual infections. However, combinations of co-infections tended to be slightly less prevalent than individual infections but still accounted for a significant proportion of cases (Table 2).

The highest prevalence of hemoparasites was identified in the 46–65-month age cohort, whereas the lowest prevalence was found in the 86–104-month cohort. For *Babesia* spp., the 66–85-month cohort exhibited the highest prevalence, with the lowest prevalence observed in the 86–104-month cohort. In the case of *Trypanosoma* spp. and *Anaplasma* spp., the highest prevalence was detected in the 66–85-month cohort, while individuals aged 106 months or older showed the lowest prevalence (Table 3).

Regarding the geographical region, the central region exhibited the highest prevalence of hemoparasites, followed by the northern, western, and southern regions. For *Babesia* spp., *Trypanosoma* spp., and *Anaplasma* spp., the central region also had the highest prevalence rates, whereas the lowest prevalence for each of these pathogens was found in the southern region (Table 4).

Similarly, the prevalence of any hemoparasite was higher in warm climates (temperatures above 24 °C) at 36.1% (130/360). The most frequently identified parasite was *Trypanosoma* spp., representing 58.7% (111/130) of cases. This was followed by *Babesia* spp. and *Anaplasma* spp., identified in 54.5% (103/130) and 54.0% (102/130) of cases, respectively. In regions with a temperate climate (between 17 and 24 °C), the prevalence of any hemoparasitic disease was 15.8% (57/360). The most prevalent species were *Trypanosoma* spp. (86.0% of cases, 49/57), followed by *Babesia* spp. (80.7% of cases, 46/57) and *Anaplasma* spp. (71.9% of cases, 41/57). At least 54.2% (195/360) of the animals presented one tick, and of these, 43.6% (85/195) were positive for *Babesia* spp. and 44.1% (86/195) for *Anaplasma* spp. Similarly, 60.8% (219/360) of the cattle were observed to have flies, of which 47.94 (105/219) were infected with *Trypanosoma* spp.

### 3.2. Association Analysis

The Pearson correlation coefficient for the prevalence of hemoparasites was positive, indicating a direct relationship between the presence of one pathogen and the manifestation of another (Table 5). The disparity ratio analysis demonstrated that 13 of the 230 variables evaluated significantly impacted the presence or absence of diseases. Cattle from the central region showed a higher risk of infection with hemoparasites than those from the southern region, while the risk was elevated in the northern and western areas. Similarly, the presence of one or more hemoparasites was significantly related to the following risk factors when compared to the control group of each variable: the weight of the animals, the purchase of animals for fattening, the disinfection of clothing after contact with neighboring animals, self-medication, the use of clean clothing, reproductive status, and the separation of pens. The following protective factors against the prevalence of any of the hemoparasitic agents were identified: the provision of mineralized salt, the presence of cattle from other owners on the farm, evidence of trauma in their joints, documented milking routines, and the sending of blood samples (Table 6).

**Figure 1:**
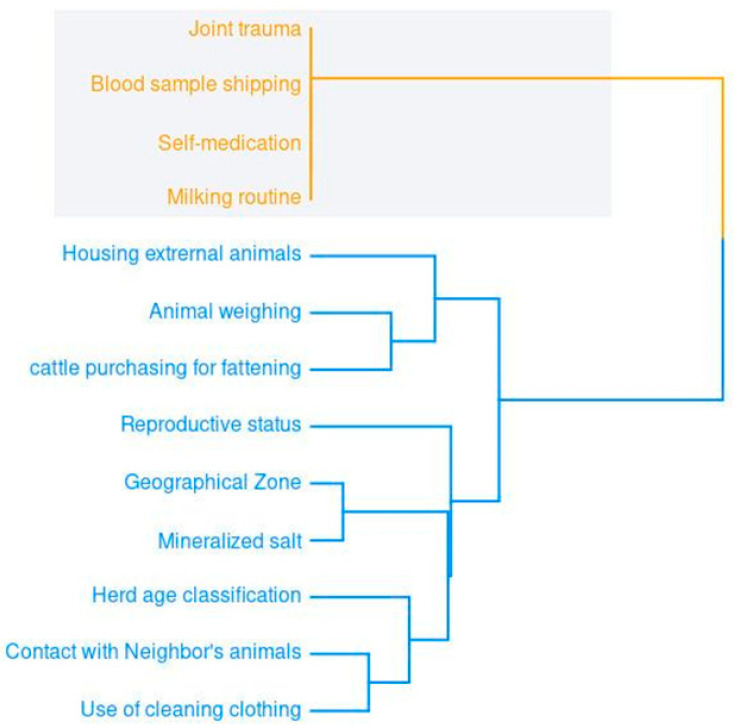
Dendrogram of hierarchical relationships among the binary variables with significant representation for hemoparasite.

The hierarchical relationships among the significant binary risk factors revealed two main groups. The first (in orange) concerns animal health and social and sanitary conditions. The second (in blue) concerns livestock activities, including commercialization, management, and biosecurity. The length of the tree branches indicates the degree of heterogeneity among the related variables (Figure 1).

## 4. Discussion

This is the first study that reveals the prevalence of hemoparasites in infectious cattle of the Andean region of southwestern Colombia associated with risk factors. This study showed that 52.5% (189/360) of cattle were infectious, showing at least one pathogen actively involved in the maintenance of endemism because the PCR technique could identify the hemoparasites in the active phase of multiplication. Consequently, the cattle without this condition have low parasite loads, and hemoparasitism must be undiagnosed [16]. The high prevalence observed in infectious cattle is related to the infestation rates with ticks and flies. *Babesia* spp. and *Anaplasma* spp. are primarily transmitted by the tick *Rhipicephalus* (*Boophilus*) *microplus*, the main vector, which is widely distributed in Colombia, including the department of Huila. In the case of *Anaplasma* spp., transmission can also occur mechanically through hematophagous dipterans. Conversely, bovine trypanosomosis is caused by vectors from the *Tabanidae* family, a type of fly with high prevalence across the department of Huila. Therefore, the same animals are uninfested during periods of high arthropod activity, so enzootic transmission is unlikely into infectious cattle. In this context, the development of immune resistance following re-infection may be limited. Conversely, higher arthropod infestation rates were associated with increased exposure to hemoparasites in the cattle herd, which could be re-infected during periods of peak infestation throughout the year [17]. Therefore, it is possible that infectious animals could develop a persistent infection, maintaining high levels of parasitism and shedding them in contact with arthropod vectors [18]. This assumption is aligned with prevalence findings identified by age, which show 34.4% and 31.9% positivity in cattle between 66–85 and 46–65 months, respectively. This suggests that these animals could be persistently infected and actively participate in the transmission cycle of hemoparasites.

Regarding the transmission cycle, the environmental conditions favor the multiplication of arthropod vectors. Previous works in other areas of the country showed that transitional periods between wet and dry seasons increase the water vapor and favor the development of the non-parasite stage of ticks [19]. Similarly, a report previously showed the highest incidence of Rhipicephalus microplis in the municipalities of Huila department located in Magdalena Valley between 0 and 1000 a.s.l [20]. These findings are aligned with the highest hemoparasite prevalence identified in the north and warm climate municipalities of the Huila Department. However, the highest prevalence level of *Trypanosoma* spp. regarding infestation involved flies showing significant participation of these arthropods in the transmission cycle, which could involve *Anaplasma* spp. Similarly, the development of flies is related to rainfall levels identified in these areas that favor the biting flies [12].

About the risk factors associated with hemoparasites in cattle, this study showed that the purchase of animals from feedlots was a risk factor for the presence of at least one pathogen. In line with this, a study in England showed that the likelihood of disease spread increased when farms purchased animals from common markets. Thus, the movement of pathogen-carrying cattle between farms facilitates the spread of disease to different areas [21]. Purchasing new animals and iatrogenic transmission practices increase the likelihood of disease spreading [22]. In this study, 58.6% (211/360) of the respondents implemented quarantine measures for cattle entry. However, only 15.3% (55/360) carried out pre-diagnosis. This situation leads to the introduction of hemoparasites as quarantine does not guarantee the detection of carriers. Similarly, in a previous study in other areas of the country, the movement of persistently infected cattle for hemoparasites increased the success of transmission within farms [3].

It has been observed that farms where animals have joint trauma tend to have lower levels of hemoparasites. This is because the most used antibiotic in these herds is oxytetracycline, known for its broad spectrum and ease of use [23]. In the search for quick fixes, farmers often resort to metaphylactic measures with readily available antibiotics to treat any trauma, infection, or decline in production. As a side effect, these treatments eliminate hemoparasites. A study conducted in Colombia found that treatment with oxytetracycline for *Trypanosoma* spp. and *Anaplasma* spp. had an efficacy rate of 100% and 75.6%, respectively [11]. They also investigated the efficacy of oxytetracycline (70 mg/mL) against *Babesia* spp. in combination with diminazene (35 mg/mL). They concluded that the treatment reduced the presence of hemoparasites in experimental cattle and restored hematological values in animals with mild parasitemia. At the same time, they observed a similar reduction in the control group, which consisted of naturally diseased cattle with severe parasitemia [24].

Similarly, 84.2% (303/360) of cattle were self-medicated, identified as a risk factor in the analysis. In Iran, oxytetracycline resistance genes were identified: otrA in 60%, otrB in 26.7%, and 13.3% in samples infected with *Anaplasma* spp. [25]. In Burkina Faso, cattle tested in five out of eight zones were found to be resistant to isometamidium chloride to treat *Trypanosoma* spp. [26]. Similarly, it has been shown in vitro that *Babesia* spp. can develop resistance to the drug diminazene acetate, especially when administered at doses lower than the recommended minimum [27]. In line with this, the indiscriminate use of different types of drugs and the lack of knowledge about the correct dosage may increase or create antimicrobial resistance in some hemoparasites, especially when self-medication is a common practice among livestock farmers. In contrast, pregnant animals were found to be at higher risk of hemoparasite infection because they were immunocompromised, and it suggested the introduction of best management practices in them.

The 400–450 kg weight was a risk determinant for some hemoparasites. This is representative because it shows the average weight of female cattle in the productive stage [28]. In the case of this study, age had no significance (*p* > 0.05) concerning the three pathogens individually and together. However, cattle over 46 months were more susceptible to hemoparasites. In Colombia, a similar study did not obtain statistical significance for age, although the prevalence was higher in cattle between 7 and 8 years old than in those of intermediate ages [29]. Internationally, cattle over 24 months were 1.5 times more susceptible to *Anaplasma* spp. than younger animals due to biological or mechanical vectors [30]. Considering the above, as the animal’s weight and age increase, there is a more significant impact on its health, which results in decreased productivity.

Mineralized salt acts as a protective factor due to the importance of micro- and macroelements. It contributes to the diet, especially when it comes to low-quality food. These nutritional elements are essential for improving disease resistance and ensuring proper physiological and reproductive functioning [31]. In the same way, mineralized salt plays a fundamental role for ruminal bacteria, essential to improving the degradation and generation of nutrients by the ruminal microbiota. The increase in the production of microorganisms benefits the immune system and helps prevent various diseases in cattle. Microbiota growth indicates a better use of nutrients and increases animal immune activity. Microelements such as zinc, manganese, selenium, and copper prevent oxidative stress, and this action improves the cellular health of animals, which, in turn, contributes to the prevention of hemoparasitic diseases [32].

Finally, good livestock practices include using appropriate and exclusive clothing for production. In this study, it was observed that clothing was a risk factor. Cases have been observed where work clothing on other farms serves as a reservoir for pathogens, which, together with poor disinfection, leads to the spread of pathogens. Washing water alone is ineffective; soaps that do not contain specific components, such as methanol or polysorbate 20, are less effective at eliminating either the vector or its eggs [33]. Besides that, washing with water, with or without surfactants, at room temperature is not sufficient to eliminate the tick [34]. A recent study concluded that a temperature of 43 °C or higher for at least 30 min in immersion is sufficient to suppress the tick [35]. Therefore, establishing preventive measures and pinpointing effective management strategies are essential to enhancing herd protection and reducing the epidemiological threat posed by hemoparasitic agents [5,8].

## 5. Conclusions

We conclude that the cattle in the study area were found to be infectious, and the herd management practices and environment favored the agents’ transmission. These findings reinforce the importance of preventive and integrated strategies to control the vectors and borne pathogens and the importance of introducing technical support for cattle management in tropical areas. Finally, it is necessary to strengthen Huila livestock through training and knowledge transfer to producers, marketers, and professionals in the sector. Investment in education and research is the key to ensuring the region’s healthier and prosperous future.

## Figures and Tables

**Table 1:** Primer sequence used for PCR amplification.

Hemoparasite	Strand	Primer Sequence 5′-3′
*Anaplasma* spp.	Forward	GTGCTGGTTGTGTGGTTGTC
	Reverse	TGGCTGGGAGGACACATACT
*Babesia* spp.	Forward	AACGTGATTTCAACAATGGTGT
	Reverse	TCTTAACCCAACTCACGTACCA
*Trypanosoma* spp.	Forward	TTGTCGTTTTCAATGGGGGA
	Reverse	GTAAAAATCACGGACGCCCC

**Table 2:** Prevalence of hemoparasites and co-infection.

Hemoparasite	Frequency	Prevalence	95% Confidence Interval	
			Lower Limit	Upper Limit
*Anaplasma* spp.	145/360	40.3	0.35	0.45
*Babesia* spp.	151/360	41.9	0.37	0.47
*Trypanosoma* spp.	162/360	45.0	0.40	0.50
*Anaplasma* spp. + *Babesia* spp.	131/360	36.4	0.31	0.41
*Anaplasma* spp. + *Trypanosoma* spp.	133/360	36.9	0.32	0.42
*Babesia* spp. + *Trypanosoma* spp.	130/360	36.1	0.31	0.41
*Anaplasma* spp. + *Babesia* spp. + *Trypanosoma* spp.	126/360	35.0	0.30	0.40

**Table 3:** Positive cases (+) for the presence of hemoparasites, *Babesia* spp., *Trypanosoma* spp., and *Anaplasma* spp., related to the age of individuals.

Age	*Hemoparasite*		*Babesia* spp.		*Trypanosoma* spp.		*Anaplasma* spp.	
	+	%	+	%	+	%	+	%
0 to 45	25	42.4	22	37.3	21	35.6	21	35.6
46 to 65	70	60.9	53	46.1	57	49.6	50	43.5
66 to 85	70	56.4	58	46.8	62	50.0	56	45.2
86 to 104	9	37.5	6	25.0	9	37.5	8	33.3
106 or more	12	40.0	9	30.0	10	33.3	7	23.3
undetermined	3	37.5	3	37.5	3	37.5	3	47.5
	χ^2^ (5, N = 360) = 11.5, *p* = 0.048		χ^2^ (5, N = 360) = 7.2, *p* = 0.208		χ^2^ (5, N = 360) = 6.71, *p* = 0.243		χ^2^ (5, N = 360) = 6.34, *p* = 0.274	

**Table 4:** Distribution of positive cases for *Babesia* spp., *Trypanosoma* spp., and *Anaplasma* spp. hemoparasites in cattle by geographic region in the department of Huila.

Region	*Hemoparasite*		*Babesia* spp.		*Trypanosoma* spp.		*Anaplasma* spp.	
	+	%	+	%	+	%	+	%
Southern	20	30.8	16	24.6	20	30.8	14	21.5
Central	43	74.1	37	63.8	36	62.1	33	56.9
Northern	96	54.2	76	42.9	78	44.1	76	42.9
Western	30	50.0	22	36.7	28	46.7	22	36.7
	χ^2^ (3, N = 360) = 23.6, *p* ≤ 0.001		χ^2^ (3, N = 360) = 20.1, *p* ≤ 0.001		χ^2^ (3, N = 360) = 6.69, *p* = 0.006		χ^2^ (3, N = 360) = 17.0, *p* ≤ 0.001	

**Table 5:** Pearson correlation for babesiosis, anaplasmosis, and trypanosomiasis.

Hemoparasites	*Babesia* spp.	*Anaplasma* spp.	*Trypanosoma* spp.
*Babesia* spp.	1	0.805 *	0.702 *
*Anaplasma* spp.	0.805 *	1	0.77 *
*Trypanosoma* spp.	0.702 *	0.771 *	1

* = statistical significance *p*-value ≤ 0.05.

**Table 6:** Binary logistic regression analysis of factors associated with the presence of hemoparasites, *Babesia* spp., *Trypanosoma* spp., and *Anaplasma* spp.

Factor		n	Hemoparasites		*Babesia* spp.		*Trypanosoma* spp.		*Anaplasma* spp.	
			*p*-Value	OR	*p*-Value	OR	*p*-Value	OR	*p*-Value	OR
Geographical area	South	65	-	-	-	-	-	-	-	-
	Center	58	<0.001 *	6.4	<0.001 *	5.4	<0.001 *	3.7	<0.001 *	4.8
	North	177	0.001 *	2.7	0.01 *	2.3	0.063	1.8	0.003 *	2.7
	West	60	0.03 *	2.2	0.145	1.8	0.069	2.0	0.064	2.1
Mineral salt supply	No	82	-	-	-	-	-	-	-	-
	Yes	269	0.005 *	0.478	0.006 *	0.5	<0.001 *	0.4	0.005 *	0.487
Livestock weight	<350	39	-	-	-	-	-	-	-	-
	351−400	66	0.397	1.4	0.668	1.2	0.183	1.8	0.83	0.9
	401−450	85	0.027 *	2.4	0.07 *	2.1	0.006 *	3.1	0.017 *	2.7
	>451	76	0.241	1.6	0.15	1.8	0.19	1.7	0.238	1.6
Presence of cattle from other owners on the farm	No	305	-	-	-	-	-	-	-	-
	Yes	55	<0.001 *	0.3	0.001 *	0.3	0.002 *	0.4	0.008 *	0.4
Purchase of animals for fattening	No	286	-	-	-	-	-	-	-	-
	Yes	74	0.035 *	1.8	0.019 *	1.8	0.006 *	2.1	0.001 *	2.9
Disinfection of clothing after contact with neighbor’s animals	No	148	-	-	-	-	-	-	-	-
	Yes	210	0.002 *	1.9	<0.001 *	2.2	0.002 *	1.9	<0.001 *	2.5
Self-medication	No	56	-	-	-	-	-	-	-	-
	Yes	303	0.017 *	2	0.001 *	3.1	<0.001 *	3.2	0.006 *	2.5
Evidence of joint trauma	No	315	-	-	-	-	-	-	-	-
	Yes	44	0.328	0.7	0.008 *	0.4	<0.001 *	0.3	0.005 *	0.23
Documented milking routine	No	264	-	-	-	-	-	-	-	-
	Yes	95	0.256	0.8	0.036 *	0.6	0.924	1	0.449	0.8
Use of clean clothing	No	109	-	-	-	-	-	-	-	-
	Yes	251	0.01 *	1.8	0.002 *	2.2	0.011 *	1.8	0.006 *	2
Blood sample submission	No	261	-	-	-	-	-	-	-	-
	Yes	98	0.049 *	0.6	0.001 *	0.4	0.034 *	0.6	<0.001 *	0.4
Reproductive status	Empty	287	-	-	-	-	-	-	-	-
	Pregnant	64	0.041 *	1.8	0.505	1.2	0.024 *	1.9	0.124	1.5
	Postparturient	8	0.484	1.7	0.232	2.4	0.659	1.4	0.176	2.7
Separation of cattle pens	No	76	-	-	-	-	-	-	-	-
	Yes	284	0.076	1.6	0.818	1	0.009 *	2.0	0.226	1.4

* = factor with significance *p*-value ≤ 0.05; - = reference factor.

## Data Availability

The authors confirm that the employed data supported the published claims, and the datasets analyzed during the study are available from the corresponding author upon reasonable request.
